# Association of decreased mitochondrial DNA content with ovarian cancer progression

**DOI:** 10.1038/sj.bjc.6603377

**Published:** 2006-10-03

**Authors:** Y Wang, V W S Liu, W C Xue, A N Y Cheung, H Y S Ngan

**Affiliations:** 1 Department of Obstetrics & Gynecology, People's Hospital, Peking University, Beijing, China; 2 Department of Obstetrics & Gynecology, Queen Mary Hospital, University of Hong Kong, Hong Kong SAR, China; 3 Department of Pathology, People's Hospital, Peking University, Beijing, China; 4 Department of Pathology, University of Hong Kong, Pokfulam Road, Hong Kong SAR, China

**Keywords:** mitochondrial DNA, copy number, ovarian carcinoma

## Abstract

Mitochondrial DNA (mtDNA) content in ovarian carcinomas was assessed by quantitative PCR. Results show that mtDNA content in tumour cell was significantly higher than that in normal ovary. Change in mtDNA content was not related with patients' age or tumour stages. However, the average mtDNA copy number in pathological low-grade tumours was over two-fold higher than that in high-grade carcinomas (*P*=0.012). Moreover, type I carcinomas also had a significantly higher mtDNA copy number than in type II carcinomas (*P*=0.019). Change in mtDNA content might be an important genetic event in the progression of ovarian carcinomas.

Mitochondrion has its own genome, the mitochondrial DNA (mtDNA). Unlike nuclear genome, mtDNA exists in each cell with several hundreds to more than 10 000 copies. Generally, mtDNA content is tissue-specific and has a steady-state level in each type of tissue. Its maintenance depends mainly on nuclear-encoded factors which usually confers function through the Tfam pathway (Moraes, 2001). Mitochondrial DNA copy number in cell is not under stringent control; and various internal or external factors associated with ATP demand may influence its level, such as: exercise (Lim *et al*, 2000), hypoxia (Hoppeler *et al*, 2003), and steroid hormones stimulation (Weitzel *et al*, 2003). It is well known that carcinoma cells proliferate fast and survive in strict microenvironment, for example, under hypoxic condition. Either down- or upregulation of mtDNA content has been observed in a number of human malignancies (Jones *et al*, 2001; Simonnet *et al*, 2002; Lee *et al*, 2004; Wong *et al*, 2004). More importantly, mtDNA content change has also been found to be associated with histological types of gastric carcinoma (Wu *et al*, 2005).

In our previous studies, we have demonstrated the occurrence of high frequencies of somatic mtDNA mutations in endometrial (Liu *et al*, 2003) and ovarian carcinomas (Liu *et al*, 2001). In addition, we have also found that mtDNA copy number was significantly elevated in endometrial adenocarcinoma when compared with normal endometrial glandular epithelium (Wang *et al*, 2005). To further determine the mtDNA content change and its relationship with cancer, we therefore investigate the mtDNA copy number in primary ovarian carcinomas.

## MATERIALS AND METHODS

Frozen samples of 38 cases of primary epithelial ovarian carcinomas and four borderline ovarian tumours diagnosed and treated from January 1991 to December 2004 were retrieved without specific selection for this study. The mean age is 52 (ranging from 26 to 83). The clinical and pathologic characteristics of the 42 patients with ovarian carcinomas are shown in Table 1. In addition, frozen samples of 17 normal ovarian tissues were also used for this study. Use of clinical samples in this study was approved by the Ethics Committee of the University of Hong Kong.

In order to obtain pure tumour cell from cancerous tissues, laser capture microdissection (LCM) was employed. The LCM procedure and DNA isolation from LCM procured samples were previously described in detail (Wang *et al*, 2005). The recipes and conditions of quantitative PCR reaction were also described in our previous study (Wang *et al*, 2005).

The raw data were processed using the software accompanying the ABI PRISM 7700 Sequencing Detection System. Linear regression was used to analyse the response of Ct (the cycle number at which fluorescence raises above the baseline level during real-time quantitative PCR) *vs* the logarithm of the DNA concentration. Pearson's correlation was used to test the relationship between patients' age and mtDNA copy numbers in tumour tissues. The copy number comparison between groups was performed by nonparametric test (Mann–Whitney or Kruskal–Wallis test). Statistical significance was set at *P*<0.05.

## RESULTS

**Figure 1:**
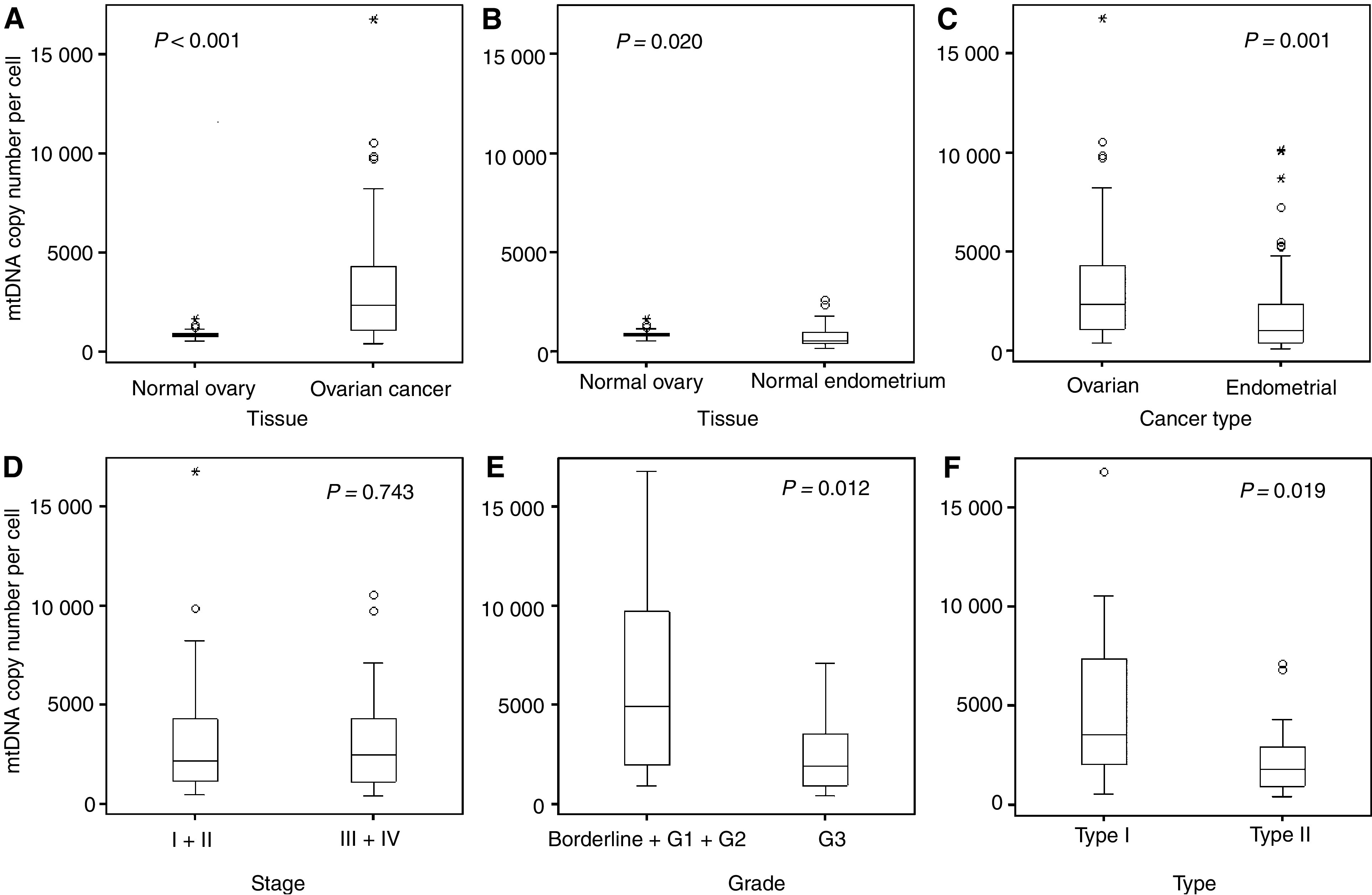
The comparisons of mtDNA copy number in cells among different groups of data. (**A**) The mean mtDNA copy number in normal ovarian cells (*n*=17) and ovarian carcinoma cells (*n*=42) are 929±276 and 3548±3421 copies per cell, respectively. A significant difference was detected (Mann–Whitney test, *Z*=−3.983, *P*<0.001). (**B**) The mtDNA copy number in normal ovarian cells was higher than that in normal endometrial cells (768±573, *n*=41) (Mann–Whitney test, *Z*=−2.332, *P*=0.020). (**C**) The mean mtDNA copy number in ovarian carcinoma cells (3548±3421, *n*=42) and endometrial carcinoma cells (2013±2317, *n*=65) were significantly different (Mann–Whitney test, *Z*=−3.311, *P*=0.001). (**D**) No significant difference of levels of mtDNA copy number between the early-stage tumour (3621±3974, *n*=20) and advanced stage tumour (3482±2923, *n*=22) was detected (Mann–Whitney test, *Z*=−0.327, *P*=0.743). (**E**) Significant difference of mtDNA copy number was observed between Grade 3 carcinomas (2361±1799, *n*=26) and the low-grade tumours (5877±4659, *n*=14) (Mann–Whitney test, *Z*=−2.495, *P*=0.012). (**F**) The mtDNA content in the type I tumours (4998±4279, *n*=19) was significantly higher than that in the type II tumours (2318±1930, *n*=21) (Mann–Whitney test, *Z*=−2.343, *P*=0.019).

Table 1 shows the mtDNA copy number per cell of the 42 ovarian cancer samples and the 17 normal ovarian tissues. Mitochondrial DNA content is significantly higher in ovarian tumours than in normal ovarian tissues (*P*<0.001) (Figure 1A). The mtDNA copy numbers in tumour tissues have no relationship with patients' ages.

The levels of mtDNA in normal ovary was higher than that in endometrium (Wang *et al*, 2005) (*P*=0.020) (Figure 1B). In ovarian tumours, an average of 3548±3421 copies of mtDNA was present in tumour cells. The highest and lowest copy numbers of mtDNA in cell were 16 772 and 444, respectively (Table 1). Compared with endometrial carcinomas (average mtDNA copy number=2012±2317 copies) (Wang *et al*, 2005), a significantly higher level of mtDNA copy number was found in ovarian carcinomas (*P*=0.001) (Figure 1C).

From the 42 patients, 20 were diagnosed as early-stage tumours, stage I or stage II; whereas 22 patients suffered from advanced carcinomas, stage III or stage IV. There is no significant difference of mtDNA copy numbers between the early-stage tumours (average at 3621±3974 copies per cell) and advanced stage carcinomas (average at 3482±2923 copies per cell) (Figure 1D).

As two patients were uninformative on the grading, the analysis was carried out in 40 patients. No significant difference was observed between the four borderline tumours and other invasive carcinomas (Mann–Whitney test, *Z*=−1.328, *P*=0.199). In the 26 cases of Grade 3 carcinomas, the mean value of mtDNA content in tumour cells was 2361±1799 copies. In the other 14 patients with lower-grade tumours (including the four borderline tumours), the mean copy of mtDNA molecules in each cell were 5877±4659 copies, it is over two-folds and significantly higher (*P*=0.012) than that in the higher-grade carcinomas (Figure 1E).

The mtDNA copy numbers among different histological subtypes of carcinomas was not significantly different (Kruskal–Wallis test, *χ*^2^=8.397, *P*=0.136, and df=5). Nevertheless, the mucinous tumours seem to have relatively higher level of mtDNA content than other subtypes. In the 42 cases, except two subjects of serous carcinoma uninformative on grading, 19 cases were classified as type I tumours and the other 21 cases were classified as type II tumour (for tumour classification, please see footnotes of Table 1). An average of 4998±4279 copies of mtDNA was detected in type I tumours; whereas, the mean copy number of mtDNA in type II tumours is 2318±1930. It is remarkable lower than that in the type I tumours (*P*=0.019) (Figure 1F).

## DISCUSSION

In this study, mtDNA content in ovarian carcinoma cells was found significantly higher than that in normal ovary. Except for a few reports, the change in mtDNA content does not associate with clinicopathological characteristics. Interestingly, in present study, we found that the mtDNA copy number in high-grade tumours is significantly lower than that in low-grade tumour. As the grade of tumour is a crucial prognostic factor, therefore, mtDNA content change might be an important genetic event in the progression of ovarian carcinoma.

Mitochondrial DNA copy number change was also found related with other human diseases. A significantly lower level of mtDNA content was observed in oocytes from women with ovarian insufficiency (May-Panloup *et al*, 2004). In addition, increased sperm mtDNA content was observed in male infertility (May-Panloup *et al*, 2003). The findings suggested that the change of mtDNA copy number was related with the impairment of cellular physiologic function. A probable explanation is that the change in mtDNA content would likely cause or be caused by the deficiencies in oxidative phosphorylation activity. Reactive oxygen species generation in mitochondria, which lead to DNA oxidative damage in cells, could thus impair cell function.

The significantly different levels of mtDNA content in different grades of tumour may be accounted for either by the downregulation in mtDNA replication in the high-grade tumours, or upregulation in mtDNA replication in low-grade tumours. Detection of many mtDNA alterations in the premalignant or preinvasive lesions indicated that mtDNA alterations could be an early genetic event in tumorigenesis (Chen *et al*, 2002; Ha *et al*, 2002; Durham *et al*, 2003). Mitochondrial DNA copy number was suggested to be increased by a feedback mechanism that compensates for the defective respiratory system owing to mutated mtDNA (Lee *et al*, 2000). So, it is possible that mtDNA copy number increased in early- and lower-grade malignancies.

On the other hand, long-term exposure to severe environmental insult such as hypoxia decreases the mitochondrial content of muscle fibres (Hoppeler *et al*, 2003). Undoubtedly, mitochondria are oxygen sensitive and the mtDNA content decrease might account for hypoxia. So, an alternative explanation of the decrease of mtDNA copy number in high-grade tumour is due to the fact that such tumour has rapid proliferation rate, and thus, survives in more severe hypoxia microenvironment leading to the downregulation of the mtDNA replication.

The most important finding in this study is the association between decreased mtDNA copy number with high grade and histological subtype of ovarian carcinoma. The histopathological phenotypes of ovarian carcinoma are complex. The epithelial-derived tumours including serous, mucinous, endometrioid, clear cell, transitional, squamous, mixed, and undifferentiated types comprise the majority of malignant tumours. Each of these histological subtypes is associated with distinct molecular genetic alterations (Shih Ie and Kurman, 2004; Bell, 2005). Based on recent clinicopathological and molecular studies, it has been proposed that surface epithelial tumours could be divided into two broad categories, type I and type II tumours. Mutations in *KRAS* and *BRAF* have been found to be common in low-grade serous and mucinous ovarian carcinomas (Gemignani *et al*, 2003; Sieben *et al*, 2004). *KRAS* belongs to *RAS* families which coding proto-oncogene that functions as a relay switch that transduces various growth signals in the cell surface to the nucleus through activation of the *RAS–RAF–MEK–ERK–MAP* kinase signalling pathway. Till now, no report connected the regulation of mtDNA replication with the genetic alterations in *RAS–RAF* pathway. As shown above, type I tumour had a significantly higher mtDNA copy number than that in type II tumour. So, whether or not a potential relationship between the genetic alterations in *RAS–RAF* pathway and mtDNA content change would be an interesting field to be explored.

On the other hand, mutations in the tumour suppressor molecule *p53* were detected in type II tumours frequently (Shih Ie and Kurman, 2004). Recent studies provided the potential mechanistic explanation. *p53* binding sequence was identified in mtDNA suggested that *p53* might be involved in the regulation of mitochondrial transcription and replication (Heyne *et al*, 2004). In addition, *p53* might also enhance the DNA replication function of mtDNA polymerase *γ* through their interaction. So, the loss of *p53* owing to the mutations in the type II tumours could result in the decrease of mtDNA replication (Achanta *et al*, 2005).

Taken together, the finding of grade and histological associated change in mtDNA copy number provide a novel insight of the role of mtDNA alterations in cancer progression. Mitochondrial DNA content in cell may be potentially used as a tool to predict prognosis. Mechanisms of mtDNA maintenance in carcinoma cells warrant further investigation.

## Figures and Tables

**Table 1:** MtDNA copy number and clinicopathological characteristics of patients

**Tissue**	**Code**	**Age**	**Diagnosis^a^**	**Stage^b^**	**Grade^c^**	**Type^d^**	**MtDNA copy number**
Tumour	OV100	49	Mucinous	I	Borderline	I	983
	OV086	68	Mucinous	I	Borderline	I	4913
	G119	75	Mucinous	III	Borderline	I	10516
	G104	58	Clear cell	I	Borderline	I	4943
	G147	31	Serous	I	G1	I	1337
	G007	64	Serous	II	G1	I	16772
	OV016	63	Mucinous	I	G1	I	8220
	OV078	42	Endometrioid	II	G1	I	936
	G087	39	Serous	I	G2	I	3539
	G215	55	Serous	III	G2	I	9702
	G213	38	Serous	IV	G2	I	6486
	OV006	26	Mucinous	I	G2	I	9845
	OV070	44	Endometrioid	II	G2	I	1976
	G209	58	Adenocarcinoma	II	G2	I	2103
	G164	64	Serous	I	G3	II	2252
	OV014	48	Serous	I	G3	II	462
	OV092	45	Serous	II	G3	II	707
	G020	57	Serous	III	G3	II	1995
	G117	51	Serous	III	G3	II	1064
	G208	39	Serous	III	G3	II	7104
	G212	45	Serous	III	G3	II	1839
	OV034	40	Serous	III	G3	II	4298
	OV074	68	Serous	III	G3	II	2925
	G110	62	Serous	IV	G3	II	6771
	G114	43	Serous	IV	G3	II	948
	OV008	48	Serous	IV	G3	II	794
	OV120	35	Serous	IV	G3	II	3735
	G120	68	Serous	III			1554
	OV004	39	Mucinous	IV	G3	I	519
	OV064	46	Endometrioid	I	G3	II	1516
	OV022	71	Endometrioid	II	G3	II	1659
	G040	72	Endometrioid	III	G3	II	1132
	OV002	36	Endometrioid	III			3816
	OV042	44	Adenocarcinoma	III	G3	II	4214
	G014	83	Adenocarcinoma	III	G3	II	2565
	G216	59	Clear cell	I	G3	I	3554
	OV032	37	Clear cell	I	G3	I	3611
	OV076	63	Clear cell	II	G3	I	2591
	OV012	54	Clear cell	III	G3	I	2415
	OV098	44	Poorly differentiated	III	G3	II	1770
	OV108	47	Poorly differentiated	II	G3	II	493
	OV110	48	Poorly differentiated	III	G3	II	444
							
Normal	Nor ov01						1640
	Nor ov02						771
	Nor ov03						943
	Nor ov04						856
	Nor ov05						1156
	Nor ov06						920
	Nor ov07						913
	Nor ov08						865
	Nor ov09						737
	Nor ov10						799
	Nor ov11						532
	Nor ov12						1367
	Nor ov13						766
	Nor ov14						809
	Nor ov15						753
	Nor ov16						705
	Nor ov17						1254

Abbreviation: mtDNA=mitochondrial DNA.

a The histological types of tumour were classified according to WHO criteria.

b The stage of each carcinoma was established according to the International Federation of Gynaecology and Obstetrics (FIGO) criteria.

c The grades of tumour were classified based on WHO criteria, Grades 1 (well differentiated), 2 (moderately differentiated), and 3 (poorly differentiated). Two carcinoma patients were uninformative on the grading.

d The types of tumour were classified based on recent studies (Shih Ie and Kurman, 2004; Bell, 2005), Type I tumours composed of mucinous carcinomas, low-grade serous and endometrioid carcinomas, and clear cell carcinomas. Type II tumours include high-grade serous and endometrioid carcinomas as well as undifferentiated carcinoma. Three adenocarcinomas were treated to be same as serous and endometrioid carcinomas.
